# Antibacterial Activity of *Rhus javanica* against Methicillin-Resistant *Staphylococcus aureus*

**DOI:** 10.1155/2013/549207

**Published:** 2013-10-08

**Authors:** Yong-Ouk You, Na-Young Choi, Sun-Young Kang, Kang-Ju Kim

**Affiliations:** ^1^Department of Oral Biochemistry, School of Dentistry, Wonkwang University, Iksan 570-749, Republic of Korea; ^2^Wonkwang Research Institute for Food Industry, Iksan 570-749, Republic of Korea; ^3^College of Education, Wonkwang University, Iksan 570-749, Republic of Korea; ^4^Department of Oral Microbiology, School of Dentistry, Wonkwang University, Iksan 570-749, Republic of Korea

## Abstract

In the present study, the leaves of *Rhus javanica* (*R. javanica*) were extracted with ethanol, and we investigated the antimicrobial activity of the ethanol extract of *R. javanica* against methicillin-resistant *Staphylococcus aureus* (MRSA). Control groups were treated with media containing 0.1% DMSO. The ethanol extract of *R. javanica* inhibited the growth of MRSA at concentrations ranging from 0.05 to 0.2 mg/mL and inhibited acid production at concentrations higher than 0.1 mg/mL (*P* < 0.05). MRSA biofilm formation was determined by scanning electron microscopy and safranin staining. The ethanol extract of *R. javanica* inhibited the formation of MRSA biofilms at concentrations higher than 0.05 mg/mL. In confocal laser scanning microscopy, high concentration (0.4–1.6 mg/mL) of *R. javanica* extract showed bactericidal effect in a dose-dependent manner. In real-time PCR analysis, *R. javanica* extract showed the inhibition of the genetic expression of virulence factors such as *mecA*, *sea*, *agrA*, and *sarA* in MRSA. Preliminary phytochemical analysis revealed the strong presence of phenolics. These results suggest that *R. javanica* may be a useful medicinal plant for inhibiting MRSA, which may be related to the presence of phenolics in the *R. javanica* extract.

## 1. Introduction

*Staphylococcus aureus* has been reported to cause many diseases, from mild skin infections to more serious invasive infections such as suppuration, abscess formation, pneumonia, and even fatal septicemia in human beings [1]. Prior to the antibiotic era, *S. aureus* was associated with 80% mortality, but the advent of the earliest antimicrobial substances, such as penicillin, contributed to a reduction in mortality [2]. It was reported that over 94% of *S. aureus* strains were susceptible to penicillin at that time. However, antibiotic resistance in *S. aureus* has rapidly developed due to the widespread use of antibiotics [2, 3]. Methicillin-resistant *Staphylococcus aureus* (MRSA) was isolated in the early 1960s [4]. MRSA is resistant to not only methicillin and other *β*-lactams but also many other antibacterial agents such as macrolides and aminoglycosides [5, 6]. MRSA is one of important causes of modern chronic infectious diseases [3, 5, 6]. One such antibiotic-resistant mechanism involves the production of *β*-lactamase or penicillin-binding protein 2a (PBP 2a) by MRSA [2]. MRSA forms biofilms on implanted medical devices such as catheters, plates, screws, artificial joints, and cardiac valves in patients [7, 8]. Biofilm formation by MRSA has been reported to increase the antibiotic resistance of MRSA [9]. MRSA metabolizes carbohydrates to produce organic acids, which can stimulate biofilm formation [10, 11]. At present, MRSA is emerging worldwide as one of the most important hospital and community pathogens due to its multidrug resistance. Therefore, new strategies are required to deal with MRSA-associated infections. Recently, scientists have focused on certain natural products with antipathogenic potential as candidates for new antibiotic substances [12, 13]. 

*Rhus javanica *(*R. javanica*) is a member of Anacardiaceae and is mainly produced in Korea, China, and Japan [14, 15]. It has been traditionally used to treat dysentery and diarrhea [15]. However, there is little scientific evidence regarding the effect of *R. javanica* on MRSA. In the course of screening for the antibacterial activities of natural products against MRSA, we recently found that extracts of *R. javanica* exhibit antibacterial activity against this pathogen. In the present study, we show that *R. javanica* has antimicrobial activity against and inhibits biofilm formation by MRSA. We also show the presence of major phytochemicals in *R. javanica*.

## 2. Materials and Methods

### 2.1. Materials

Brain heart infusion (BHI) broth was purchased from Difco Laboratories (Detroit, MI, USA). Glucose and dimethyl sulfoxide (DMSO) were obtained from Sigma Co. (St. Louis, MO, USA). MRSA ATCC 33591 was purchased from the American Type Culture Collection (ATCC; Manassas, VA, USA). 

### 2.2. Plant Material and Extraction

The leaves of *R. javanica* were obtained from the oriental drug store Dae Hak Yak Kuk (Iksan, South Korea). The identity of the specimen was confirmed by Dr. Bong-Seop Kil at the Department of Natural Science, Wonkwang University (Iksan, South Korea). A voucher specimen (number 05-11) has been deposited at the Herbarium of the Department of Oral Biochemistry in Wonkwang University. Dried leaves (600 g) of *R. javanica* were soaked in 3000 mL of 90% ethanol for 72 h at room temperature. The extracted solution was filtered and evaporated under reduced pressure to yield an ethanol extract of 42.4 g (7.1%). After the extract was thoroughly dried to facilitate complete removal of the solvent, the dry extract was dissolved in DMSO to give the desired stock solution. The final concentration of DMSO applied to culture systems was adjusted to 0.1% (v/v), which did not interfere with the testing system. Control groups were treated with media containing 0.1% DMSO. 

### 2.3. Bacterial Growth and Acid Production

Bacterial growth was determined using a modification of a previously described method [16, 17]. The growth of MRSA was examined at 37°C in 0.95 mL of BHI broth containing various concentrations of the ethanol extract of *R. javanica*. These tubes were inoculated with 0.05 mL of an overnight culture grown in BHI broth (final: 5 × 10^5^ colony-forming units (CFU)/mL), and incubated at 37°C. After 24 h of incubation, the optical density (OD) of cells was measured spectrophotometrically at 550 nm, and the pH of the cultures was determined using a pH meter (Corning Inc., Corning, NY, USA). Three replicates were measured for each concentration of the test extract. NaF (1%) was used as a positive control. 

### 2.4. Biofilm Assay

The biofilm assay was based on a method described previously [18, 19]. *R. javanica* extract was added to BHI broth containing 1% glucose in 35 mm polystyrene dishes (Nunc, Copenhagen, Denmark). The cultures were then inoculated with a seed culture of MRSA (final: 5 × 10^5^ CFU/mL). After cultivating for 48 h at 37°C, the supernatant was removed completely, and the dishes were rinsed with distilled H_2_O. The biofilm formed on the surface of the dishes was also stained with 0.1% safranin, and photographed.

### 2.5. Scanning Electron Microscopy (SEM)

 The biofilm on 35-mm polystyrene dishes was also determined by SEM using a modification of a previously described method [20]. The biofilm formed on the dishes was rinsed with distilled H_2_O and fixed with 2.5% glutaraldehyde in 0.1 M sodium cacodylate buffer (pH 7.2) at 4°C for 24 h. After gradual dehydration with ethyl alcohol (60%, 70%, 80%, 90%, 95%, and 100%), the sample was freeze-dried. The specimens were then sputter-coated with gold (108A sputter coater, Cressington Scientific Instruments Inc., Watford, England, UK). For observation, a JSM-6360 SEM (JEOL, Tokyo, Japan) was used.

### 2.6. Bactericidal Effect of *R. javanica* Extract

 Bactericidal effect of *R. javanica* extract was determined by confocal laser scanning microscopy. The cultured MRSA in BHI was diluted using BHI media to approximately 1 × 10^7^ CFU/mL. The bacteria (1 × 10^7^ CFU/mL) were treated with high concentrations (0.2–1.6 mg/mL) of *R. javanica* extract at 37°C under aerobic conditions. After 30 min of incubation, the bacteria were washed with PBS and stained with LIVE/DEAD BacLight Bacterial Viability Kit (Molecular Probes, Eugene, OR, USA), prepared according to the manufacturer's instructions, for 15 min. Stained bacteria were observed confocal laser scanning microscopy (LSM 510, Zeiss, Germany). This method is based on two nucleic acid stains: green fluorescent SYTO 9 stain and red fluorescent propidium iodide stain which differ in their ability to penetrate healthy bacterial cells. SYTO 9 stain labels live bacteria, in contrast propidium iodide penetrates only bacteria with damaged membranes. 

### 2.7. Real-Time Polymerase Chain Reaction (PCR) Analysis

To determine the effect of *R. javanica* extract on gene expression, a real-time PCR assay was performed. The sub-minimal inhibitory concentration (0.01–0.1 mg/mL) of *R. javanica* extract was used to treat and culture MRSA for 24 h. Total RNA was isolated from *S. mutans* by using Trizol reagent (Gibco-BRL) according to the manufacturer's instructions. Then, cDNA was synthesized using a reverse transcriptase reaction (Superscript; Gibco-BRL). The DNA amplifications were carried out using an ABI-Prism 7,000 Sequence Detection System with Absolute QPCR SYBR Green Mixes (Applied Bio systems Inc., Foster City, CA, USA). The primer pairs that were used in this study were described by previous reports [21–23] and are listed in Table 1. 16S rRNA was used as an internal control. 

### 2.8. Phytochemical Screening

 Phytochemical tests of the extract were performed as previously described [24, 25]. Mayer's reagent was used for alkaloids, ferric chloride reagent for phenolics, Molish test for glycosides, Biuret reagent for peptides, Mg-HCl reagent for flavonoids, Libermann-Burchard reagent for steroids, and silver nitrate reagent for organic acids. 

### 2.9. Statistical Analysis

 All experiments were performed in triplicate. Data were analyzed using the Statistical Package for Social Sciences (SPSS, Chicago, IL, USA). The data are expressed as the mean ± standard deviation values. The differences between the means of the experimental and control groups were evaluated by Student's *t*-test. Values of *P* < 0.05 were considered statistically significant.

## 3. Results 

### 3.1. Bacterial Growth Inhibition by *R. javanica*

**Figure 1:**
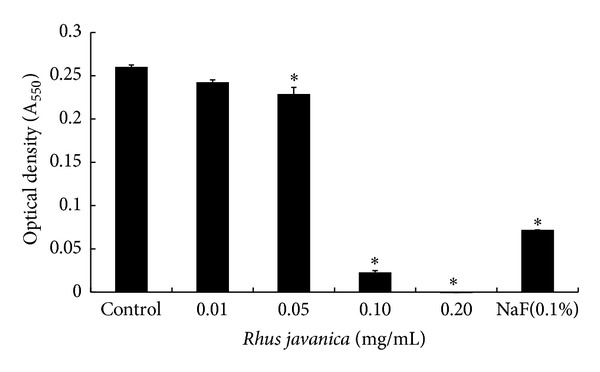
Effect of ethanol extract of *R. javanica* on the growth of MRSA. MRSA was inoculated into BHI broth with various concentrations of *R. javanica* and incubated for 24 h at 37°C. The optical density (*A*_550_) was read using a spectrophotometer. Data are mean ± standard deviation. *P < 0.05 compared to the control group.

In the present study, we investigated the antibacterial activity of the extract of *R. javanica* against MRSA. The bacteria were exposed to 0.01, 0.05, 0.1, and 0.2 mg/mL of the ethanol extract of *R. javanica*. As seen in Figure 1, the extract (0.05–0.2 mg/mL) showed antibacterial activity against MRSA in a dose-dependent manner and resulted in high MRSA growth inhibition at concentration >0.1 mg/mL compared to the control group (*P* < 0.05). The positive control (0.1% NaF) also indicated antibacterial activity. The minimum inhibitory concentration (MIC) for the ethanol extract of *R. javanica* is 0.1 mg/mL. The determination of the MIC revealed the antimicrobial activity of the ethanol extract of *R. javanica* against MRSA. 

### 3.2. Inhibitory Effect of *R. javanica* on Acid Production

 Acid productions of the bacteria treated with *R. javanica* extract were also monitored. As summarized in Table 2, there was an obvious decrease in pH in the control group, but the decrease was substantially inhibited in the presence of the extract (0.1–0.2 mg/mL). The decrease in pH was also inhibited in the positive control group (0.1% of NaF). These results demonstrate that *R. javanica* extract may inhibit organic acid production in MRSA. 

### 3.3. Inhibitory Effect of *R. javanica* on Biofilm Formation

**Figure 2:**
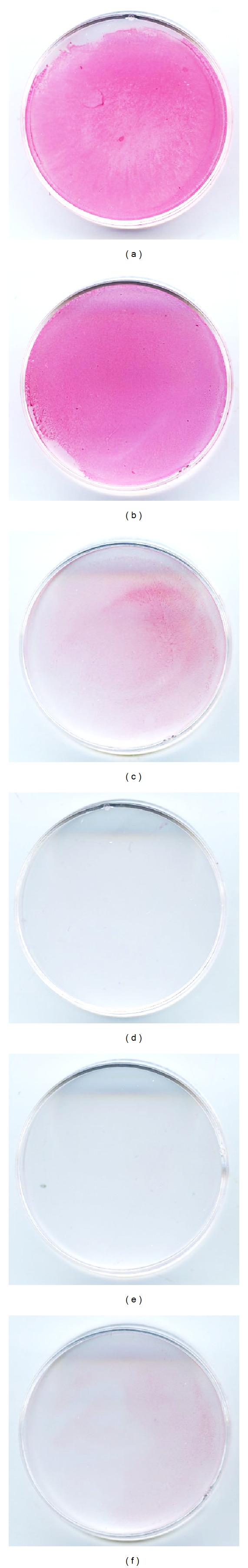
Effect of ethanol extract of *R. javanica* on biofilm formation by MRSA. MRSA was inoculated into BHI broth with various concentrations of *R. javanica* and incubated for 48 h at 37°C. The biofilm formed on the surface of the dishes was also stained with 0.1% safranin and photographed. (a) Control; (b) 0.01 mg/mL; (c) 0.05 mg/mL; (d) 0.1 mg/mL; (e) 0.2 mg/mL; (f) positive control (0.1% NaF).

**Figure 3:**
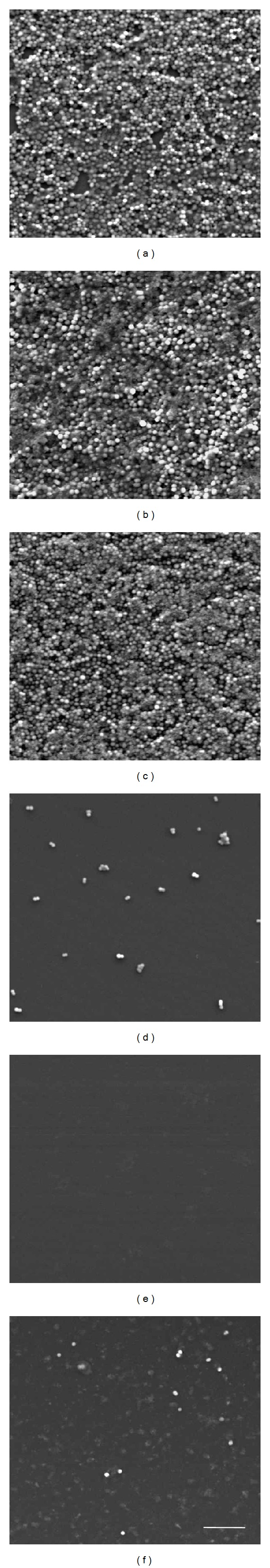
Scanning electron microscopy of MRSA biofilms grown in ethanol extract of *R. javanica*. (a) Control; (b) 0.01 mg/mL; (c) 0.05 mg/mL; (d) 0.1 mg/mL; (e) 0.2 mg/mL; (f) positive control (0.1% NaF); bar = 10 *μ*m.

 We examined the inhibitory effect of *R. javanica* extract on MRSA biofilm formation by safranin staining. As shown in Figure 2, the extract of *R. javanica* (0.05–0.2 mg/mL) inhibited biofilm formation by MRSA. Biofilm formation was also inhibited in the presence of the positive control (0.1% NaF). SEM photographs underline the results obtained by safranin staining (Figure 3). MRSA attached to and aggregated on the surface of polystyrene 35 mm dishes and visibly formed the biofilm in the control group, but biofilm formation was lower in the presence of *R. javanica* extract at concentrations higher than 0.05 mg/mL. Biofilm formation was also lower in the presence of the positive control.

### 3.4. Bacteriocidal Effect of *R. javanica*

**Figure 4:**
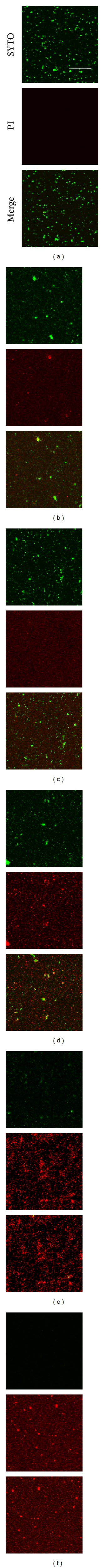
Bactericidal effect of ethanol extract of *R. javanica*. Cultured MRSA was treated with high concentration (0.2–1.6 mg/mL) of *R. javanica* extract and stained with LIVE/DEAD BacLight Bacterial Viability Kit. The stained bacteria were observed by confocal laser scanning microscopy. Treatment with ethanol extract of *R. javanica* decreased green-labeled living bacteria (SYTO 9 stain) and increased red-labeled dead bacteria (PI stain) in a dose-dependent manner. (a) Control; (b) 0.2 mg/mL; (c) 0.4 mg/mL; (d) 0.8 mg/mL; (e) 1.6 mg/mL; (f) positive control (0.1% NaF); bar = 50 *μ*m.

Bactericidal effect of *R. javanica* extract was tested by confocal laser scanning microscopy (Figure 4). Bacterial viability was gradually decreased at high concentration (0.4–1.6 mg/mL) of *R. javanica* extract in a dose-dependent manner. 

### 3.5. Inhibitory Effect of *R. javanica* on Virulence Factor Gene Expression

**Figure 5:**
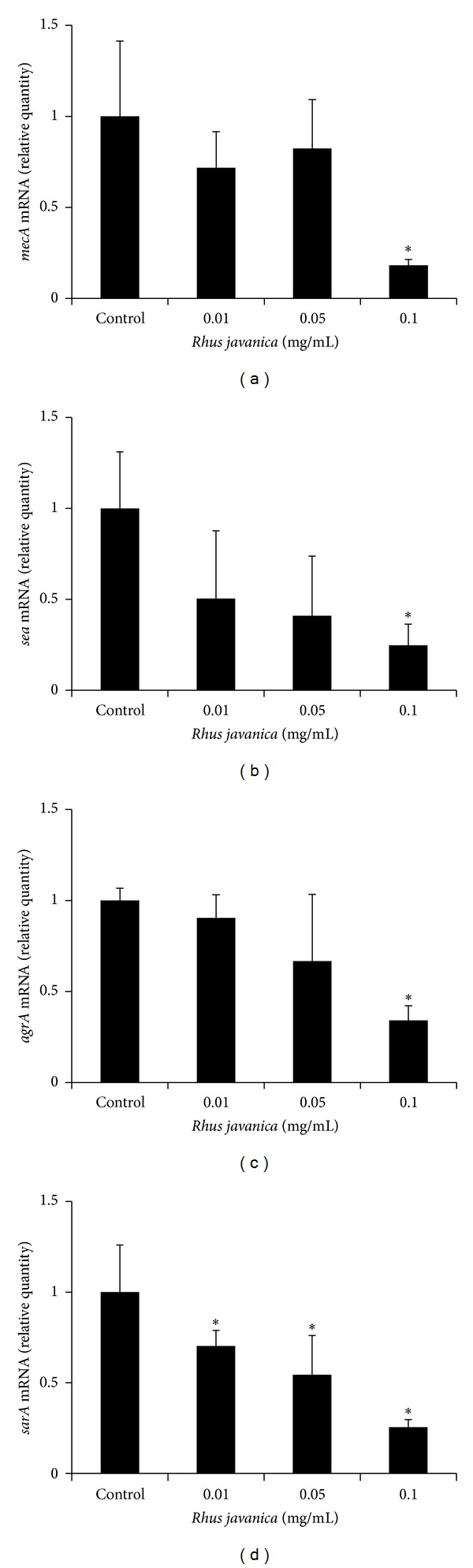
Real-time PCR analysis of expression of several virulence factor genes. MRSA was cultured and treated with sub minimal inhibitory concentration (0.01–0.1 mg/mL) of *R. javanica* extract, and real-time PCR analysis was then performed as described in Section 2.  *mecA*, *sea*, and *agrA *expression was significantly inhibited at 0.1 mg/mL of *R. javanica* extract, and *sarA *was significantly inhibited at concentration higher than 0.01 mg/mL. Each value is expressed as a mean ± standard deviation. Significance was determined at *P < 0.05 when compared with the control.

 Real-time PCR analysis was performed to examine the effect of *R. javanica* extract on the genetic expression of virulence factors in MRSA (Figure 5). The expression of *mecA*, *sea*, *agrA*, and* sarA *was significantly decreased in MRSA (*P* < 0.05) when it was treated with *R. javanica* extract.

### 3.6. Phytochemical Analysis

The results of the phytochemical tests conducted on the ethanol extract of *R. javanica* are shown in Table 3. Preliminary phytochemical analyses revealed the strong presence of phenolics, the moderate presence of glycosides, and the weak presence of flavonoids, steroids (terpenoids), and organic acids. These results suggest that phenolics may have been responsible for the antibacterial activity observed in the present study.

## 4. Discussion

In the present study, we examined the antimicrobial activity of *R. javanica* extract against MRSA. Our data show that 0.05–0.2 mg/mL of the ethanol extract of *R. javanica* inhibited the growth of MRSA. The fact that the extract of *R. javanica* inhibited MRSA growth provides a scientific rationale for the use of this extract by local inhabitants as an antimicrobial agent. Traditionally, *R. javanica* has been used to treat dysentery and diarrhea in Korea, China, and Japan [15].

Previous studies have shown that MRSA is able to metabolize dietary carbohydrates and thereby produce organic acids [10, 11]. The major organic acid produced by MRSA is acetic acid, which can lower the pH of infected regions and contribute to the formation of firmly adhering biofilm-like microbial communities [10]. In the present study, the extract of *R. javanica* inhibited the decrease of pH induced by MRSA. This result suggests that the extract of *R. javanica* may inhibit carbohydrate metabolism in MRSA. 

MRSA has the ability to adhere, colonize, and form biofilms on damaged tissue, implanted medical devices, and prosthetic devices [7, 8]. Biofilms are surface-associated bacterial communities on biological or abiotic substrata. They are enclosed firmly in a self-produced extracellular matrix composed of polysaccharides and proteins. Biofilms are very difficult to remove and are a source of refractory infections. Biofilm formation on the surface of implanted medical devices frequently requires surgical removal of the biofilm, debridement of the surrounding tissue, and long-term antibiotic treatment [7, 8]. The most well-known assay method for the detection of biofilm formation is the tissue culture plate assay method [9]. In our study, the extract of *R. javanica* inhibited biofilm formation by MRSA at concentrations ranging from 0.05 to 0.1 mg/mL, as seen by safranin staining. SEM data of MRSA biofilm formation were consistent with the data from safranin staining. However, the data on biofilm culture were different from the results of planktonic cell growth measurements. The growth of MRSA in planktonic culture was inhibited at concentrations higher than 0.05 mg/mL. A previous study has shown that bacteria in biofilm cultures are physiologically different from planktonic cells of the same organism. Biofilm formation enhances bacterial resistance to both the host defense system and antimicrobials [26]. However, in our study, bacterial resistance against *R. javanica* may be similar within both biofilm cultures and planktonic cultures.

Bactericidal effect of *R. javanica* extract was tested by confocal laser scanning microscopy. Bacterial viability was gradually decreased at high concentration (0.4–1.6 mg/mL) of *R. javanica* extract in a dose-dependent manner. This result suggests that high concentration of *R. javanica* extract may be bactericidal on MRSA.

An antibiotic resistance gene, *mecA* encodes PBP2a, which has low affinity to *β*-lactam antibiotics, so it allow cell-wall biosynthesis despite the presence of *β*-beta-lactams [2]. In the present study, the effect of *R. javanica* extract on the genetic expression of *mecA *was determined by real-time PCR analysis. The expression of *mecA *was significantly decreased in MRSA when it was treated with *R. javanica* extract.

A virulence factor gene, *sea *encodes Staphylococcal enterotoxin A which is one of major virulence factors in MRSA [27]. Staphylococcal enterotoxin A is one cause of gastroenteritis in human and acts as a superantigen. In this study, the *R. javanica* extract significantly inhibited *sea* expression. *sea *gene expression in MRSA is regulated by global regulators such as *agr* and *sarA* gene [21]. In our study, *R. javanica* extract showed the inhibition of *agrA* and* sarA* expressionin MRSA. *agrA *encodes accessory gene regulator A which positively regulates exotoxin-encoding genes. *sarA* also upregulates expression of virulence factor genes. Previous research has shown that inhibition of *agrA* or* sarA* expression by some chemicals such as thymol or clindamycin reduces transcription of exotoxin-encoding genes [21]. In the present study, suppressive effect of *R. javanica* extract on *sea *gene expression may, in part, be related with inhibitory effect of *R. javanica* extract on *agrA* and* sarA* expression [28].

Previous reports indicate that the leaves of *R. javanica *contain mainly tannins (50–70%) such as tannic acid, pyrogallol, gallic methy lester, syringic acid, protocatechuic acid, and 1,2,3,4,6-penta-*O*-galloyl-*β*-d-glucose [14, 29, 30]. In the present study, we found the strong presence of phenolics, the moderate presence of glycosides, and the weak presence of flavonoids, steroids (terpenoids), and organic acids. These results suggest that phenolics may have been responsible for the antibacterial activity observed in the present study. However, further studies are needed to elucidate the antimicrobial principles underlying the action against MRSA.

In conclusion, our study demonstrated the antimicrobial activity of the ethanol extract of *R. javanica* against MRSA. *R. javanica* inhibited the growth, acid production, and biofilm formation of MRSA. *R. javanica* also showed bactericidal effect and the inhibition of the genetic expression of virulence factors such as *mecA*, *sea*, *agrA*, and *sarA*. Phytochemical analysis revealed the strong presence of phenolics. These results suggest that *R. javanica* may be a useful medicinal plant for inhibiting MRSA, which may be related to the presence of phenolics in the *R. javanica *extract. 

## Figures and Tables

**Table 1:** Nucleotide sequences of primer used for real-time PCR in this study.

Gene	Gene description	Primer sequences (5′-3′)	
16S rRNA	Normalizing internal standard	Forward	ACTGGGATAACTTCGGGAAA
		Reverse	CGTTGCCTTGGTAAGCC

*mecA *	Penicillin binding protein 2′	Forward	GTTAGATTGGGATCATAGCGTCATT
		Reverse	TGCCTAATCTCATATGTGTTCCTGTAT

*sea *	Staphylococcal enterotoxin A	Forward	ATGGTGCTTATTATGGTTATC
		Reverse	CGTTTCCAAAGGTACTGTATT

*agrA *	Accessory gene regulator A	Forward	TGATAATCCTTATGAGGTGCTT
		Reverse	CACTGTGACTCGTAACGAAAA

*sarA *	Staphylococcal accessary regulator A	Forward	TGTTATCAATGGTCACTTATGCTG
		Reverse	TCTTTGTTTTCGCTGATGTATGTC

**Table 2:** Effect of ethanol extract of *R. javanica* on acid production by MRSA.

Concentration (mg/mL)	pH (before incubation)	pH (after incubation)
Control	7.20 ± 0.05	5.80 ± 0.05^1^
0.01	7.13 ± 0.05	5.13 ± 0.20
0.05	7.13 ± 0.04	5.73 ± 0.36*
0.10	7.13 ± 0.05	6.67 ± 0.23*
0.20	7.13 ± 0.00	7.17 ± 0.40*
NaF (0.1%)	7.20 ± 0.00	7.18 ± 0.20*

^1^Data (pH) are represented as mean ± standard deviation. *P < 0.05 compared to the control group after incubation.

**Table 3:** Phytochemical analysis of the ethanol extract of *R. javanica. *

Plant constituents	Ethanol extract
Alkaloids	−
Phenolics	+++
Flavonoids	+
Glycosides	++
Peptides	−
Steroids, terpenoids	+
Organic acids	+

+++: strong; ++: moderate; +: weak; −: absent.
